# Influence of Training Load on Mood Disturbance at Sea Level and 3900 m Altitude: A Case Study of a Wheelchair Athlete

**DOI:** 10.3390/sports6040122

**Published:** 2018-10-22

**Authors:** Santiago Sanz-Quinto, Gabriel Brizuela, Raúl López-Grueso, Ian Rice, Manuel Moya-Ramón

**Affiliations:** 1 Sport Research Centre, Miguel Hernandez University, 03202 Elche, Spain; atleta@santiago-sanz.com (S.S.-Q.); raul.lopezg@umh.es (R.L.-G.); 2 Department of Physical and Sports Education, University of Valencia, 46010 Valencia, Spain; Gabriel.brizuela@uv.es; 3 Department of Kinesiology and Community Health College of Applied Health Sciences, University of Illinois at Urbana-Champaign, Champaign, IL 61820, USA; ianrice@illinois.edu; 4 Department of Health Psychology, Miguel Hernandez University, Elche, Institute for Health and Biomedical Research (ISABIAL-FISABIO Foundation), 03010 Alicante, Spain

**Keywords:** hypoxic environment, POMS, athletics, paralympic, baroreflex sensitivity

## Abstract

The purpose of this case study was to investigate the influence of a training load (TL), oxygen saturation (SO_2_) and blood pressure (BP) on mood states in a wheelchair marathoner during (7 weeks at sea level (SL), 5 weeks at 3860 m altitude, 1 week returning to SL). TL was obtained with Foster’s equation while mood states were obtained with the Profile of Mood States Questionnaire (POMS). Furthermore, SO_2_ and BP were assessed upon wakening. SO_2_ (%) decreased at altitude, compared to SL (88.31 ± 2.46 vs. 98.52 ± 0.11) and increased until the last week at altitude (92.64 ± 1.12). Systolic pressure (SP) increased at altitude compared to pre-altitude (126.0 ± 5.1 vs. 107.6 ± 4.4 mmhg), and was not different from the last week at altitude. Controlling for SO_2_ and SP, differences were also observed in fatigue (97.66 ± 18.92 vs. 17.39 ± 13.71) and vigor (73.23 ± 8.62 vs. 26.48 ± 11.89) as a function of altitude. Upon return to SL, fatigue, vigor, SO_2_ and SP returned to pre values. This case study demonstrated the POMS was sensitive to worsening patterns in fatigue and vigor at altitude through a practical survey approach combined with daily physiological assessment.

## 1. Introduction

The Profile of Mood States questionnaire (POMS) [1] reflects the individual mood in six primary dimensions (Depression-Dejection, Tension-Anxiety, Anger-Hostility, Vigor-Activity, Fatigue-Inertia, Confusion-Bewilderment) and is widely used in sports to evaluate the psychological state of athletes. High values on the vigor-activity scale and low values in the remaining scales are desirable for athletic performance and resemble an iceberg formation when plotted. Exercise has been suggested to alter mood, depending on the type, volume, duration and intensity [2]. An optimal balance between training and rest may prevent overtraining symptoms, which have been characterized by fatigue, performance decrements, mood changes, irritability, and loss of motivation [3]. Moreover, during periods of overtraining, athletes generally report undesirable changes in Total Mood Disturbance (TMD), which represents the sum of the five negative scales of POMS, subtracting vigor score and adding a constant of 100 [4]. Therefore, flattened or even inverted iceberg profiles on the POMS subscales have been observed in non-functional, overreaching, or over trained athletes [5], as high values on the vigor-activity scale and low values in the remaining scales are desirable for athletics performance. Furthermore, when training load (TL) is reduced during taper periods, athletes have reported improvements in mood as reflected by a return of POMS scores to baseline. Moreover, it has been suggested that a 50% or greater increase in an individual’s basal off-season TMD score may be reflective of an overreaching state [6]. 

Numerous researchers have reported an increase in negative mood states at altitudes above 3050 m [7]. For example, in a recent publication where the POMS was administered to a nine person team ascending 6000 m during a Mount Everest expedition, the authors reported oscillations in fatigue and vigor during the ascent; however, the depression scale was unaffected [8]. More specifically, upon psychological evaluation of the athletes using the POMS, mood changes are most severe during the first or second day at altitude and then gradually recede over the next 2–4 days [9]. 

To the best of our knowledge, use of the POMS to assess elite marathoners is absent from the literature, despite the fact that marathon running has been shown to affect mood states more negatively than low to moderate intensity exercise [10]. Therefore, it may be reasonable to surmise that similar mood disturbance may be precipitated by a combination of intense marathon training performed under normoxic and hypoxic environments. However, to date, there is a dearth of literature pertaining to mood response of wheelchair athletes in training scenarios at SL or hypoxic conditions. One exception, found elite athletes with and without disabilities possessed similar iceberg profiles and thus, had superior emotional health compared to the general population, implying “elite” rather than “ability” was most influential to mood status [11]. Moreover, it must be considered that only one study has assessed the physiological-psychological response of a Paralympic athlete under altitude conditions [12]. In fact, recently, the same study reported a disturbance in oxygen saturation (SO_2_) and brachial blood pressure (BP) upon arrival to altitude; however, to our knowledge there are no studies that have analyzed the influence of these physiological variables on mood states.

Despite the aforementioned finding, the extent to which wheelchair athletes experience mood responses similar to able-bodied athletes is largely unknown. Therefore, the purpose of this study was to investigate the psychological response of an elite wheelchair marathoner to a TL under both, normoxic and hypoxic conditions. Furthermore, we examined the influence of SO_2_, systolic and diastolic blood pressure (SP and DP) on mood states, when these physiological variables were added as covariates. To achieve this goal, our study was divided into three aims:

Aim 1—To examine the psychological response of an elite-wheelchair marathoner during a TL at sea level (SL), considered baseline.

Aim 2—To examine how the same athlete and TL performed at altitude (3860 m) and how this impacts psychological response.

Aim3—To determine if any changes in mood state occurring at altitude would return back to baseline when training resumed at SL under a reduced TL.

## 2. Materials and Methods

### 2.1. Participant

The athlete was a 36 year-old, professional wheelchair marathoner, International Paralympic Committee (IPC) class T52 (wheelchair athletes with arm involvement), multiple times world champion, Paralympic Games silver medalist, 13 world records; height = 1.76 m; Body Mass = 52.6 ± 0.4 kg; VO_2max_ = 52 mL kg^−1^ min^−1^; VO_2_ at second ventilatory threshold (VT2) = 48 mL kg^−1^ min^−1^ training 8000 km per year. The athlete was familiarized with altitude training camps set at 1600–2900 m in the last 13 years with next models: live-high-train-high (LHTH), live-high-train-low (LHTL) and high-high-low (LHTHTL) [13]. Altitude camp was performed as a preparation for Boston and London marathons, where he finished first with a course record and second respectively in his division. The participant provided written informed consent for participation and the study was conducted in accordance with the Declaration of Helsinki, and the protocol was approved by the Ethics Research Committee of the University (project identification code # DPS.MMR.02.15).

### 2.2. Procedures

#### 2.2.1. Training Features at Sea Level and Altitude

**Figure 1:**
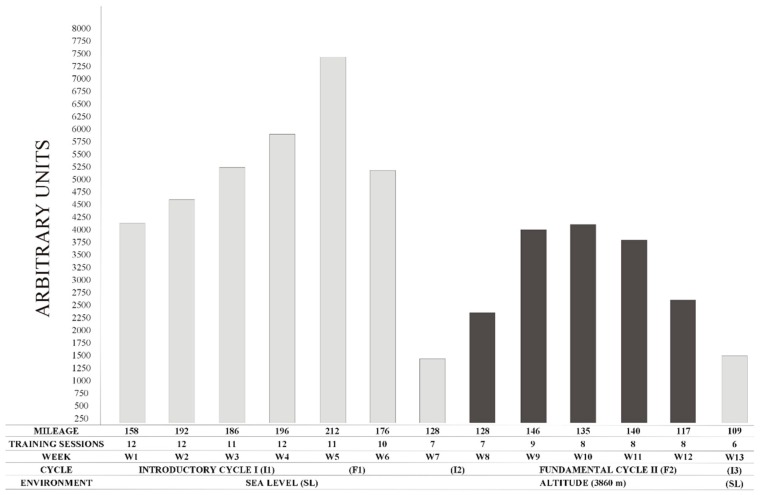
Weekly Training Load.

Our study occurred between SL at 16 m altitude at the Peruvian Altiplano (Puno, 3860 m altitude). The study period occurred from 23 November 2015 to 21 February 2016 (13 weeks; 91 days), and was composed of the athlete’s introductory cycle I (I1) (weeks 1 to 4) at SL, where training contents included arm ergometer workouts, over ground Nordic ski sessions and workouts below the first ventilatory threshold (<VT1) with the racing chair. Fundamental cycle I (F1) (weeks 5 and 6) occurred at SL where training included arm ergometer workouts, over ground Nordic ski, long moderate workouts at approximately VT1, two interval sessions at approximately VT2, and strength sessions in the gym. Introductory cycle 2 (I2) (weeks 7 and 8) occurred at SL week 7 and shifted to 3860 m altitude in week 8, where the athlete performed the same 16 to 20 km workouts <VT1 during the pre-altitude and acclimatization week. Fundamental cycle II (F2) (weeks 9 to 12) at 3860 m altitude, involved only intense sessions if the athlete reached a reference value of his Heart Rate Variability (HRV), considered as optimal to perform intense sessions, explained in detail previously [11]. The Introductory cycle 3 (I3) (week 13) occurred at SL, as post-altitude, and involved training identical to I2 (See Figure 1).

#### 2.2.2. Mood States Assessment and Training Load Calculation

Mood states were assessed using the Spanish short form version of the Profile of Mood States self-report questionnaire (POMS). This version consists of 59 adjectives; each rated on a five-point scale. From week 1, every Sunday (rest day) and after breakfast, our athlete responded to the question of how he had felt during the past week including that day. First week results constituted baseline 1. Week 7 data was not included for analysis because it coincided with overseas travel. 

Physical TL was defined with Foster’s equation [14] as the product of the rating of perceived effort (RPE), using Borg scale 1–10 (where 1 means nothing at all and 10 extremely strong) [15] multiplied by the active time of the session duration. RPE was recorded 30 minutes after the morning and afternoon sessions and was calculated based on morning sessions (1-day training) or as the average of the morning and afternoon (two session training day). Daily TL was calculated by summing the Fosters values for the morning and afternoon sessions. Weekly TL was calculated as the summation of daily values from Monday to Saturday (See Figure 1).

#### 2.2.3. Oxygen Saturation and Brachial Blood Pressure Assessment

The SO_2_ was measured with a finger pulse oximeter (Colson 650 2100, Frouard, France) in a seated position upon waking.

Brachial blood pressure (BP) was measured in a seated position, with the validated (Omron HEM-705CP, OMRON HEALTHCARE, INC., Lake Forest, IL, USA) oscillometric sphygmomanometer. Measurements were made in triplicate and averaged. Both systolic (SP) and diastolic (DP) blood pressure were recorded as well. 

The assessment, results and discussion of both SO_2_ and BP have been previously reported [12].

### 2.3. Statistical Analysis

Kolmogorov-Smirnov was used to assess data normality. A repeated measures ANOVA was performed for POMS dimensions with TIME at two levels (SL and altitude). Oxygen saturation (SO_2_), systolic blood pressure (SP) and diastolic blood pressure (DP) were added as covariates. A post hoc LSD multiple range test determined the differences between factor levels. Pearson’s correlation coefficients were calculated for tension, fatigue, vigor, anger, depression, confusion, TMD, weekly Foster, SO_2_, SP and DP. Effect size (d) associated with change in every POMS dimension was calculated using Cohen’s d (difference in mean scores over time divided by pooled SD) and was interpreted as trivial (≤0.19), small (0.20–0.49), medium (0.50–0.79), and large (≥0.08) [16]. Statistical significance was set at alpha = 0.05. Statistical analyses were performed using SPSS version 22.0 (SPSS, Inc., Chicago, IL, USA) software.

## 3. Results

Results can be seen in Table 1 and Table 2.

### 3.1. POMS

We observed significant differences between SL and altitude in two dimensions of the POMS (fatigue and vigor), while considering SO_2_ and SP as covariates. Fatigue was significantly higher at altitude compared to SL (97.66 ± 18.92 vs. 17.39 ± 13.71, *p* = 0.0362, d = 6); also, vigor was significantly lower at altitude (73.23 ± 8.62 vs. 26.48 ± 11.89, *p* = 0.0484, d = 6.1). No significant differences were observed between SL and altitude in any POMS dimension when DP was considered as the covariate. 

### 3.2. Oxygen Saturation

The SO_2_ improved with altitude exposure from acclimatization until the fifth week of exposure (88.31 ± 2.46 vs. 92.64 ± 1.12, *p* = 0.001, d = 2.7), although it remained stable at SL (98.08 ± 0.26 to 98.77 ± 0.14). 

### 3.3. Brachial Blood Pressure

SP remained significantly higher (*p* = 0.001) at altitude compared to SL. Values were maximal during the third week of exposure (132.4 ± 3.4) and reached their lowest magnitude by the last altitude week (124.9 ± 3.5). Upon returning, SL become significantly lower compared to the last altitude week (113.1 ± 3.1, *p* = 0.001, d = 2). A deeper report on SO_2_ and BP during the experiment can be found in Reference [12]. 

**Figure 2:**
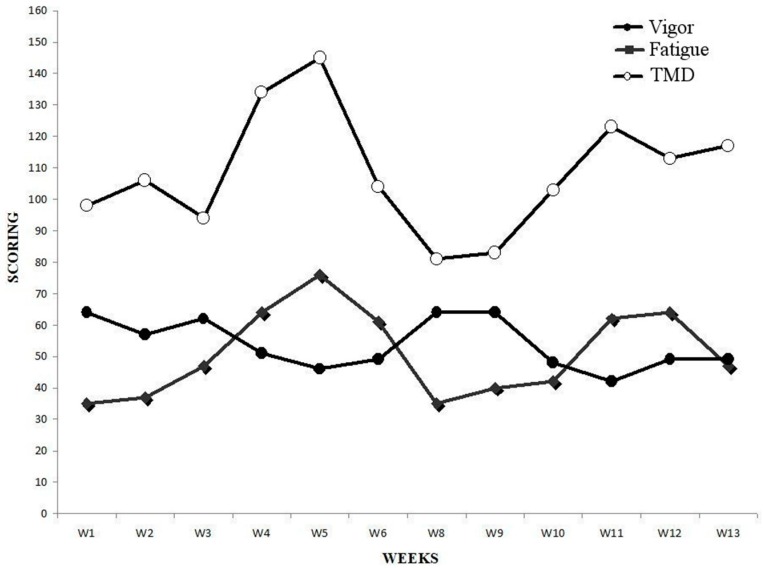
Weekly POMS vigor, POMS fatigue and total mood disturbance.

Oscillations in POMS dimensions can be found in Figure 2. 

### 3.4. Total Mood Disturbance

We observed the greatest values in TMD in week 5 (145 A.U.) and week 4 (134 A.U.), where weekly Foster reached its greatest level (7406.86 A.U. in week 5 and 5916.1 A.U in week 4). Indeed, a strong correlation was observed between TMD and weekly Foster (r = 0.66; *p* = 0.0258). In week 5, when SO_2_ remained high at SL, value for fatigue reached the greater level (76 A.U.), and so did anger (56 A.U.) which was not influenced by SO_2_, SP and DP when considered as covariates. Furthermore, we found a strong correlation between fatigue and anger (r = 0.70; *p* = 0.0109). Both depression and confusion remained similarly stable during the entirety of the experiment. However, in the last week, confusion increased 38.7% and the magnitude of change compared to the last altitude week was large (d = 3.7). Moreover, we found a strong correlation between depression and confusion (r = 0.60; *p* = 0.0367). Finally, tension was greater pre-altitude and a strong correlation was found between tension and weekly mileage (r = 0.67; *p* = 0.0244).

## 4. Discussion

The purpose of this study was to investigate the psychological response of an elite wheelchair marathoner to a TL under both normoxic and hypoxic conditions. In addition, we examined the influence of SO_2_, SP and DP on the psychological response, through their addition as covariates. Finally, we examined if mood changes occurring from SL to altitude would resolve to baseline levels when training resumed at SL under reduced TL.

Consistent with previous studies performed on able-bodied athletes at altitude ranging from 3080 m to 6000 m [7,8], we found increased fatigue and decrease vigor with altitude. In addition, we observed that increased TL at SL had a negative effect on fatigue and TMD, which peaked in week five when TL was maximal. These findings resemble those of elite swimmers who showed decreased vigor after 1 day of a 2/3 volume increase and elevated fatigue and overall mood scores after 2 days of increased TL [17]. Interestingly, TMD in the current study peaked in week 5, although training volumes in weeks four and five were similar (196.4 vs. 211.6 km), which may suggest a Foster’s threshold was reached and might significantly affect TMD. In fact, a TMD 48% greater than baseline, may be reflective of overreaching symptoms [6]. It seems that altitude had a lower impact on TMD than a greater volume of training performed at SL; however, we must consider that training volume at altitude was 1/3 lower compared to SL. Moreover, an inverted iceberg profile occurred in weeks 5 and 12, which again may signify overreaching symptoms [5]. Similarly, we found confusion and TMD were negatively affected upon return to SL where levels exceeded those observed during pre-altitude 1. Despite a strong positive correlation between tension and mileage, high tension scores occurred in the first 2 weeks, which may reflect stress imposed by equipment preparation for the season. Finally, we observed that vigor and fatigue returned to baseline levels upon return to SL.

A limitation of the current study was to find in the current literature, similar experimental designs among the elite marathoners population; and to the best of our knowledge, only one study with middle distance collegiate athletes has been set at similar altitude (4000 m) [18]. Other possible limitations might be the influence of loneliness on mood states at weeks four and five at altitude, however, the athlete feedback on social relations with the hotel’s workers and area coaches was quite positive. Finally, this study was also limited with the influence that variables such as poor wheelchair accessibility in town and hotel might have on tension and anger dimensions. A positive aspect regarding training places was perfect tarmac, where athlete had no incidences, other than one flat tire.

## 5. Conclusions

In conclusion, our study shows that despite the implementation of only one questionnaire per week, compared to daily physiological variables assessment [12], POMS was sensitive to worsening patterns in fatigue and vigor at altitude, which might be influenced by a disturbance in SO_2_ and SP. Furthermore, effects on fatigue and TMD may be more pronounced at SL once TL reaches its peak, in an elite wheelchair marathoner. Fortunately, the POMS is non-invasive, easy to assess, inexpensive, and has excellent internal consistency and reliability ((Cronbach’s α) range from 0.84 to 0.95) [1]. Therefore, we encourage its use for coaches and athletes interested in monitoring oscillations in psychological response to TLs during training at varying altitudes.

Often times, altitude training camps must be completed by athletes in remote locations, without direct assistance from coaches, physicians, doctors or psychologists. This study shows that a wheelchair athlete could self-monitor TL with simple and non-expensive devices, along with the use of a weekly mood profile assessment (POMS), to better convey his (physiological-psychological) state to technical staff during a training program under greater stressful conditions, as a challenging environment. Our research may help to inform the design and power of future studies performed under similar conditions. Future studies should continue to examine greater numbers of elite wheelchair marathon racers with more diverse disability characteristics (paraplegics, tetraplegics, spina bifida, etc.) to replicate and validate our findings.

## Figures and Tables

**Table 1:** Weekly POMS dimensions values and rate of perceived exertion.

	Tension	Fatigue	Confusion	Depression	Anger	Vigor	TMD	BORG
W1	55	35	31	39	39	64	98	4
W2	57	37	31	39	42	57	106	4
W3	37	47	31	39	39	62	94	5
W4	55	64	33	41	51	51	134	5
W5	54	76	33	39	56	46	145	6
W6	29	61	31	40	38	49	104	5
W7	72	64	60	44	76	48	182	4
W8	29	35	31	39	39	64	81	4
W9	28	40	31	39	39	64	83	5
W10	28	42	33	43	46	48	103	6
W11	35	62	33	41	48	42	123	5
W12	34	64	31	39	44	49	113	5
W13	40	47	43	42	46	49	117	4

TMD = total mood disturbance; W1 to W7 and W13, weeks at sea level; W8 to W12, weeks at altitude (3860 m). W7 data was not included for analysis because it coincided with overseas travel; BORG, is reported as the integer value from the weekly average of morning and afternoon sessions.

**Table 2:** Weekly oxygen saturation, systolic and diastolic blood pressure.

	SO_2_ (%)	SP (mmHg)	DP (mmHg)
W1	98.33 ± 0.25 ^†‡§¶^**	113.8 ± 4.3 ^†‡§¶^**	72.6 ± 3.1 *^†‡§¶^
W2	98.77 ± 0.14 ^†‡§¶^**	117.9 ± 3.6 ^†‡§¶^**	77.4 ± 3.9 *^†‡§¶^
W3	98.11 ± 0.42 ^†‡§¶^**	115.4 ± 6.1 ^†‡§¶^**	75.1 ± 4.5 *^†‡§¶^
W4	98.56 ± 0.24 ^†‡§¶^**	112.7 ± 9.1 ^†‡§¶^**	71.4 ± 3.9 *^†‡§¶^
W5	98.74 ± 0.17 ^†‡§¶^**	112.3 ± 6.8 ^†‡§¶^**	70.9 ± 5.8 *^†‡§¶^
W6	98.52 ± 0.11 ^†‡§¶^**	107.6 ± 4.4 ^†‡§¶^**	68.6 ± 3.8 *^†‡§¶^
W7	98.64 ± 0.14 ^†‡§¶^**	111.0 ± 3.3	68.4 ± 3.3
W8	88.31 ± 2.46 *	126.0 ± 5.1	80.4 ± 5.2 *
W9	91.19 ± 0.76 ^†^	127.1 ± 4.8 *	81.1 ± 3.9 *
W10	91.92 ± 0.82 ^†^	132.4 ± 3.4 *	83.4 ± 4.1 *
W11	92.35 ± 1.14 ^†‡^	125.7 ± 6.9 *	80.0 ± 2.1 *
W12	92.64 ± 1.12 ^†^	124.9 ± 3.5 *^§^	77.7 ± 2.1 *^§^
W13	98.08 ± 0.26 ^†‡§¶^**	111.3 ± 7.6 ^†‡§¶^**	73.7 ± 4.7 *^†‡§¶^

SO_2_ = oxygen saturation; SP = systolic blood pressure; DP = diastolic blood pressure. * Differences from W7 (*p* < 0.01); ^†^ Differences from W8 (*p* < 0.01); ^‡^ Differences from W9 (*p* < 0.01); ^§^ Differences from W10 (*p* < 0.01); ^¶^ Differences from W11 (*p* < 0.01); ** Differences from W12 (*p* < 0.01).
